# Redox-Active Molecules as Therapeutic Agents

**DOI:** 10.3390/antiox11051004

**Published:** 2022-05-20

**Authors:** Ana Sofia Fernandes

**Affiliations:** CBIOS, Universidade Lusófona’s Research Center for Biosciences & Health Technologies, Campo Grande 376, 1749-024 Lisbon, Portugal; ana.fernandes@ulusofona.pt

Oxidative stress and altered redox signaling have been described in a plethora of pathological conditions, such as inflammation, cardiovascular diseases, diabetes, cancer, and neurodegenerative disorders, among others [1]. The concept of redox-active therapeutics explores the potential usefulness of redox-active molecules to modulate the etiology/progression of such diseases. Although the therapeutic potential of many natural and synthetic compounds has been suggested for decades, recent advances in molecular biology and pharmacology have strengthened this field of research by providing novel mechanistic insights, especially regarding the redox modulation of critical signaling pathways. The scope of this Special Issue is to give a broad and updated overview of the therapeutic potential of redox-active molecules, covering from fundamental science to clinical research, focused on the potential effects of either natural or synthetic compounds on different redox-related diseases.

Redox-modulating strategies have been widely explored in the cancer pharmacology field. Some classical chemotherapeutic drugs, such as doxorubicin, are known to increase intracellular ROS levels [2]. A review paper by Mirzaei et al. [3] addresses the role of nuclear factor erythroid 2-related factor 2 (Nrf2) signaling in doxorubicin resistance. Furthermore, the modulation of Nrf2 as a strategy to ameliorate the side effects of doxorubicin is discussed. Another combinational therapy is proposed in a research paper by Manguinhas et al. [4], in a study on non-small-cell lung cancer cells. The authors explored the combination of cisplatin with E3330, an inhibitor of the redox function of the apurinic/apyrimidinic endonuclease 1. This compound was able to increase cytotoxicity and impair cell migration and invasion, boosting cisplatin’s anti-cancer effects. An emerging class of drugs for anticancer therapy are the inhibitors of lysyl oxidase enzymes. Ferreira et al. [5] reviewed the role of LOXL2, a member of this family of enzymes, on cancer development and metastases, with a special focus on breast cancer. The recent advances in the development of LOXL2 inhibitors are also described.

Along with synthetic drugs, many natural compounds have shown noteworthy results in cancer pharmacology. Yu et al. [6] investigated the effects of Withanolide C in breast cancer cells. The authors found that the compound exerts oxidative stress-mediated cytotoxicity, apoptosis and DNA damage in breast cancer cell lines. Another natural product with anticancer properties is the antibiotic Thiostrepton. Nelson et al. [7] explored the mechanistic basis for the interaction of Thiostrepton with peroxiredoxin 3, which is the molecular target of this drug. Plant (poly)phenols have also demonstrated anticancer activities in various models of neoplasia. Ossikbayeva et al. [8] suggest that the combination of curcumin and carnosic acid synergistically suppresses the proliferation of metastatic prostate cancer cells, and they describe the underlying mechanisms.

Besides oncology, other therapeutic areas may benefit from redox interventions. A review article from Scammahorn et al. [9] describes the current research of therapeutic strategies based on H_2_S, which displays powerful antioxidant properties, against renal and cardiovascular pathologies. Di Luigi et al. [10] proposes that the phosphodiesterase type 5 inhibitor sildenafil could be a therapeutic candidate for systemic sclerosis treatment, as it protects against oxidative damage in human dermal fibroblasts isolated from patients. In addition to small molecules, redox-active interventions may also include cell therapy products. Oxidative stress is a major cause of damage to the quantity and quality of embryos produced in vitro. A research paper by Ra et al. [11] studied the conditioned medium of amniotic membrane-derived mesenchymal stem cells as a novel antioxidant intervention for assisted reproduction.

This Special Issue also includes a clinical study carried out by Angiolillo et al. [12]. The authors evaluated the effects of *Lippia citriodora* leaf extract on lipid and oxidative blood profile of volunteers with hypercholesterolemia and suggested that dietary supplementation with such an extract could be beneficial in this condition. In fact, plants constitute an incredible and still underexplored reservoir of molecules with potential therapeutic applications. Menezes et al. [13] developed a strategy combining metabolomics, statistics, and the evaluation of (poly)phenols’ bioactivity using a yeast-based discovery platform to allow the bioprospection of natural sources of (poly)phenols with therapeutic potential for redox-related diseases.

Disturbances in glutathione homeostasis are implicated in several diseases. Therefore, different approaches aimed at replenishing glutathione levels have been suggested. The compound I-152 combines two pro-GSH molecules, *N*-acetyl-cysteine and cysteamine. Crinelli et al. [14] explored the molecular mechanisms of I-152 and demonstrated that not only does it supply GSH precursors, but it also activates the Nrf2 and the activating transcription factor 4 signaling pathways. Another novel antioxidant approach consists of selenium enrichment of yeasts and lactic acid bacteria, which combines the beneficial effects of these microorganisms and of selenium supplementation. A research paper by Krausova et al. [15] studied the bioavailability and effects of Se-enriched strains in a rat model.

This Special Issue has highlighted the vast possibilities of redox-active interventions. However, in most cases, many questions still need to be answered during the drug development journey, before these molecules could reach clinical use. The articles published in this Special Issue represent some more steps in this direction. I would like to acknowledge all the authors for their contributions.
